# Expression of the VEGF and angiopoietin genes in endometrial atypical hyperplasia and endometrial cancer

**DOI:** 10.1038/sj.bjc.6601194

**Published:** 2003-08-26

**Authors:** C M Holland, K Day, A Evans, S K Smith

**Affiliations:** 1 Department of Pathology, University of Cambridge, Tennis Court Road, Cambridge CB1 1QP, UK; 2 Department of Obstetrics and Gynaecology, University of Cambridge, The Rosie Hospital, Robinson Way, Cambridge CB2 2SW, UK

**Keywords:** VEGF-B, angiopoietins, endometrial cancer, endometrial hyperplasia, *in situ* hybridisation

## Abstract

Angiogenesis is critical for the growth and metastasis of endometrial cancer and is therefore an important therapeutic target. Vascular endothelial growth factor-A (VEGF-A) is a key molecule in angiogenesis, but the identification of related molecules and the angiopoietins suggests a more complex picture. We investigated the presence of transcripts for VEGF-A, VEGF-B, VEGF-C, VEGF-D, Angiopoietin-1 and Angiopoietin-2 in benign endometrium, atypical complex hyperplasia (ACH) and endometrioid endometrial carcinoma using *in situ* hybridisation. We confirmed the presence of VEGF-A mRNA in the epithelial cells of cancers examined (13 out of 13), but not in benign endometrium or ACH. We also demonstrate, using quantitative polymerase chain reaction, that levels of VEGF-B mRNA are significantly lower in endometrial cancer than benign endometrium. We conclude that loss of VEGF-B may contribute to the development of endometrial carcinoma by modulating availability of receptors for VEGF-A.

Angiogenesis is a critical event in the growth and spread of tumours (Folkman, 1990). The vascular endothelial growth factor family (VEGFs) and the angiopoietins are key factors in this process (Fong *et al*, 1995; Shalaby *et al*, 1995; Ferrara *et al*, 1996; Maisonpierre *et al*, 1997; Thurston *et al*, 1999). Vascular endothelial growth factor-A is a dimeric glycoprotein existing as four main isoforms by alternative splicing from a single gene. The coding regions of the VEGF gene comprise eight exons. The first four are conserved but alternative splicing of the last four gives rise to mRNAs of 121, 145, 165, 189 and 206 amino acids, respectively (VEGF_121_, VEGF_145_, VEGF_165_, VEGF_189_, VEGF_206_) (Houck *et al*, 1991; Tischer *et al*, 1991; Charnock-Jones *et al*, 1993). The VEGF-A isoforms bind the tyrosine kinase receptors VEGFR-1 (flt-1) and VEGFR-2 (KDR/flk-1) to promote endothelial cell proliferation, migration and increased vascular permeability. Elevated levels of VEGF-A are found in many tumours including cancers of the endometrium (Doldi *et al*, 1996; Fujimoto *et al*, 1998) and ovarian epithelium (Olson *et al*, 1994; Boocock *et al*, 1995; Abu-Jawdeh *et al*, 1996). When VEGF-A signalling is blocked with VEGF-neutralising antibodies (Olson *et al*, 1996) or dominant-negative receptors (Millauer *et al*, 1994), tumour angiogenesis and growth are impaired.

However, the family of VEGF genes has expanded with the discovery of other VEGFs, for example, VEGF-B (Olofsson *et al*, 1996a), VEGF-C (Joukov *et al*, 1996) and VEGF-D (Achen *et al*, 1998), which share significant sequence homology with VEGF-A. VEGF-B exists as two splice variants, a heparin binding isoform of 167 amino acids (VEGF-B_167_) and a secreted isoform of 186 amino acids (VEGF-B_186_) (Olofsson *et al*, 1996b). Whereas VEGF-A binds to both the VEGFR-1 and VEGFR-2 receptors, VEGF-B binds only to the VEGFR-1 receptor (flt-1) (Olofsson *et al*, 1998). Vascular endothelial growth factor-B forms homodimers and can heterodimerise with VEGF-A (Olofsson *et al*, 1996a) to promote endothelial cell proliferation *in vitro* (Olofsson *et al*, 1996a).

Both VEGF-C and VEGF-D are ligands for VEGFR-2 (KDR) and the third VEGF receptor VEGFR-3 (flt-4) (Joukov *et al*, 1996; Achen *et al*, 1998). VEGFR-3 is expressed by adult lymphatic endothelial cells but not by adult vascular endothelium. There is a strong association between lymphovascular density and VEGF-C expression in human malignant mesothelioma, suggesting this pathway as an important therapeutic target (Ohta *et al*, 1999).

A second family of growth factors, the angiopoietins, are ligands for the tyrosine kinase receptor Tie-2 (Davis *et al*, 1996; Maisonpierre *et al*, 1997). The two ligands share 60% sequence homology and bind with equal affinity to Tie-2. Angiopoietin-1 (Ang-1) maintains pericyte/endothelial cell interactions (Hanahan, 1997; Thurston *et al*, 1999) while Angiopoietin-2 (Ang-2) is a functional antagonist of Ang-1 and leads to marked vessel regression in tumours in the absence of VEGF-A (Holash *et al*, 1999). Compared to Ang-1, which is widely expressed in adult tissues, Ang-2 is selectively expressed at sites of active angiogenesis, for example, the corpus luteum of the ovary (Goede *et al*, 1998) and endometrium (Li *et al*, 2001).

Angiogenesis is an important feature of endometrial cancer as microvessel density is an independent prognostic indicator of both progression and overall survival (Kaku *et al*, 1997). Furthermore, there is a progressive increase in microvessel density from benign endometrium through the cancer precursor, atypical complex hyperplasia (ACH) to invasive disease (Abulafia *et al*, 1995).

While VEGF-A mRNA and protein are present in endometrial cancer (Doldi *et al*, 1996; Guidi *et al*, 1996; Fujimoto *et al*, 1998), its expression in ACH is unknown. Vascular endothelial growth factor-C (Hirai *et al*, 2001) and VEGF-D (Yokoyama *et al*, 2003) have both been correlated with a poor prognosis in endometrial cancer, but the expression of VEGF-B has not been described. In order to further investigate a possible role for other angiogenic factors in endometrial cancer, we investigated the presence of transcripts for VEGF-A, VEGF-B, VEGF-C, VEGF-D, Ang-1 and Ang-2 in benign postmenopausal endometrium, ACH and endometrioid endometrial carcinoma by *in situ* hybridisation and used quantitative polymerase chain reaction (PCR) to determine the levels of VEGF-B and Ang-2 mRNA in benign and malignant endometrium.

## MATERIALS AND METHODS

### Patients

A total of 13 carcinoma specimens were examined using *in situ* hybridisation. All were endometrioid-type carcinomas. Five benign postmenopausal endometrial specimens and five complex atypical hyperplasias were also examined for comparison. The age range of the patients was 51–87 years (median 57 years). Histopathological findings and clinical data are shown in Table 1

**Table 1:** Clinical and histopathological details for patient samples used in the study

**Patient**	**Histology**	**Age**	**Status**	**FIGO stage**	**Grade**	**LVS involved**	**Tamoxifen use**
1	Benign	51	Alive	—	—	—	N
2	Benign	57	Alive	—	—	—	N
3	Benign	80	Alive	—	—	—	N
4	Benign	63	Alive	—	—	—	N
5	Benign	68	Alive	—	—	—	N
6	ACH	56	Alive	—	—	—	N
7	ACH	58	Alive	—	—	—	N
8	ACH	52	Alive	—	—	—	N
9	ACH	69	Alive	—	—	—	N
10	ACH	52	Alive	—	—	—	N
11	Endometrioid cancer	41	Alive	1C	1	N	N
12	Endometrioid cancer	56	Alive	2B	2	N/R	N
13	Endometrioid cancer	48	Alive	2B	2	Y	N
14	Endometrioid cancer	46	Alive	1B	2	N	N
15	Endometrioid cancer	71	Alive	3A	3	N/R	N
16	Endometrioid cancer	87	Alive	1B	3	N	N
17	Endometrioid cancer	54	Alive	1B	3	N	Y
18	Endometrioid cancer	78	Deceased	1C	3	N	N
19	Endometrioid cancer	84	Deceased	4A	3	Y	N
20	Endometrioid cancer	56	Alive	3B	3	N/R	N
21	Endometrioid cancer	52	Alive	1B	3	N/R	Y
22	Endometrioid cancer	68	Deceased	4A	3	Y	Y
23	Endometrioid cancer	81	Alive	1B	3	Y	N

ACH=atypical complex hyperplasia; LVS=lymphovascular space involvement N/R=not reported N=never, Y=yes.

. Presence or absence of lymphovascular space involvement is indicated, where reported, in the table. The survival status for each patient was noted at the time of preparation of this manuscript. Ethical committee approval was obtained from the committee of Addenbrookes Hospital NHS Trust.

### Tissue samples

*In situ* hybridisation and immunohistochemistry were performed on formalin-fixed, paraffin-embedded endometrial tissue. Tissue used for reverse transcription–PCR (RT–PCR) and quantitative PCR was snap-frozen from endometrium removed during endometrial curettage or total hysterectomy, after examination by a histopathologist.

### Production of radiolabelled riboprobes for *in situ* hybridisation

Antisense and sense (control) RNA probes were generated against VEGF-A, VEGF-B, VEGF-C, VEGF-D, angiopoietin-1 and angiopoietin-2 (Sowter *et al*, 1997). For VEGF-A, probes were designed to span the first four exons thus detecting the four commonly occurring isoforms. The sequences were amplified in pCRscript SK (Stratagene Ltd., Cambridge, UK) and pBluescript II KS vectors (Stratagene Ltd., Cambridge, UK). Radioactive, single-stranded probes incorporating ^33^P-UTP (Amersham International plc, Little Chalfont, Buckinghamshire, UK) were carried out with 1 *μ*g plasmid template using the Ambion MAXIscript transcription kit (Ambion Inc., Austin, TX, USA) before phenol extraction and ethanol precipitation.

### Identification of mRNA encoding VEGF-A, VEGF-B, VEGF-C, VEGF-D, angiopoietin-1 and angiopoietin-2 by *in situ* hybridisation

Tissue sections (6 *μ*m) were cut onto slides coated with 3-aminopropyltriethoxy-silane (Sigma Chemical, Poole, Dorset, UK). The hybridisation procedure was performed as described by Clark *et al* (1996). The riboprobe was incubated with the tissue sections for 18 h at 55°C. The sections were washed and treated with RNAse A (Sigma Chemicals, St Louis, USA), before being coated with autoradiographic emulsion (Amersham Pharmacia Biotech UK Ltd, Aylesbury, UK) and exposed for 21 days at 4°C. Sections were developed with photographic developer (Kodak D19) and counterstained with Mayer's haemalum (BDH Chemicals, Lutterworth, UK) before viewing.

### Immunohistochemistry for cytokeratin and CD-68

Immunohistochemistry was performed on serial sections to identify the cell types expressing the transcripts. Mouse antibodies to human cytokeratin (clone MNF116; Dako, Ltd., High Wycombe, Buckinghamshire, UK) and human CD68, a macrophage marker, (clone PG-1; Dako, Ltd.) were used as described previously (Clark *et al*, 1996). Mouse IgG (Dako, Ltd.) incubated with serial sections provided negative controls.

### Reverse transcription–PCR

**Figure 1:**
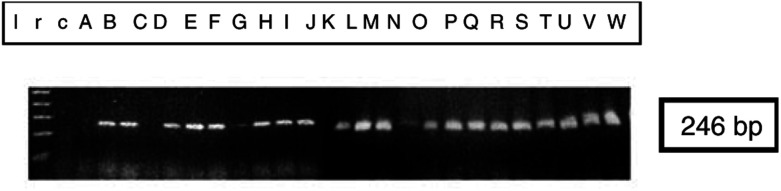
Reverse transcription PCR analysis of VEGF-C in samples of benign endometrium (A–G), ACH (H–J) and endometrioid endometrial carcinoma. Well differentiated carcinoma (K–M), moderately differentiated carcinoma (N–S) and poorly differentiated carcinoma (T–W). Lane r represents an RNA control in which the RT step was omitted and lane c represents a negative control in which the cDNA was replaced by water. The size of the transcripts (shown on the right of the figure) was estimated by co-electrophoresis of a 1 kb ladder (lane l).

RNA from benign postmenopausal endometrium, ACH and endometrioid endometrial carcinoma was analysed by RT–PCR to determine whether mRNA for VEGF-C was present (Figure 1

). Total RNA was extracted from snap-frozen tissue using acid phenol purification (Chomczynski and Sacchi, 1987). Samples for PCR were unrelated to those used for *in situ* hybridisation. RNA was annealed with oligo-dT primers (Pharmacia Biotech LTD., St Albans, UK) and cDNA was synthesised using reverse transcriptase (HT Biotechnology LTD., Cambridge, UK). Polymerase chain reaction for VEGF-C was performed using the upstream primer 5′-TGCCGATGCATGTCTAAACT-3′, and the downstream reverse complement primer 5′-TGAACAGGTCTCTTCATCCAGC-3′ for 40 cycles (15 s at 95°C and 60 s at 60°C).

### Quantitative PCR

RNA was extracted and purified and cDNA was prepared as above. Quantitative, real-time PCR was performed in triplicate using the ABI PRISM 7700 Sequence Detector (Applied Biosystems, Warrington, UK) according to the manufacturer's instructions and the resultant data were averaged. Non-template controls were included in each experiment. Specific oligonucleotide primers and probes were designed for VEGF-B using Primer Express® 1.5 software (Applied Biosystems, Warrington, UK). Specific primers and probe for Ang-2 were a gift from Mrs K Day of The Reproductive Molecular Research Group at the University of Cambridge Department of Obstetrics and Gynaecology. Details for VEGF-B primers and probe are shown below.

Forward primer, 5′–AGCACCAAGTCCGGATG–3′

Reverse primer, 5′–GTCTGGCTTCACAGCACTG–3′

Probe, 5′-6FAM–AGATCCTCATGATCCGGTACCCGT–3′

A 1 *μ*l volume of each cDNA was amplified using PCR Master Mix (Applied Biosystems, Warrington, UK) under the following PCR conditions: 50°C for 2 min, 95°C for 10 min, followed by 40 cycles of 95°C for 15 s and 60°C for 1 min. *β*-Actin and 18 s RNA were used as endogenous control sequences for VEGF-B and Angiopoietin-2, respectively. Preliminary experiments confirmed that the endogenous control sequences were amplified at the same rate as the target sequences (data not shown). The threshold cycle (*C*_T_) was determined for each sample. *C*_T_ value is the cycle number at which the level of reporter fluorescence rises above a preset threshold level. Normalisation was achieved by subtracting the mean *C*_T_ value for each endogenous control triplicate from the mean *C*_T_ value for each target gene triplicate. Target gene abundance was calculated for each tissue type.

### Statistical analyses

The presence or absence of specific *in situ* hybridisation for each transcript was compared across the different histological categories using the two-tailed Fisher's exact probability test. This was computed using the *R* statistical computing programme (www.r-project.org). Statistical significance was accepted as *P*<0.05. For results of quantitative PCR, normalised log-transformed transcript levels were compared between groups using the unpaired Student's *t*-test (two-tailed).

## RESULTS

### Expression of members of the VEGF family and angiopoietins by *in situ* hybridisation

The presence of VEGF-A was detected in all cases of endometrioid endometrial cancer (Table 2

**Table 2:** Expression of VEGF-A, VEGF-B, VEGF-C, VEGF-D, Ang-1 and Ang-2 detected by *in situ* hybridisation in sections of endometrium

Endometrial histology	*n*	VEGF-A	VEGF-B	VEGF-C	VEGF-D	ANG-1	ANG-2
Benign	5	0/5	0/5	0/5	0/5	1/5	0/5
ACH	5	1/5	5/5*	0/5	3/5	1/5	1/5
FIGO G1/2 carcinoma	4	4/4*	3/4	0/4	1/4	0/4	0/4
FIGO G3 carcinoma	9	9/9*	4/9	0/9	5/9	0/9	4/9

* *P*<0.05.

and Figure 2

**Figure 2:**
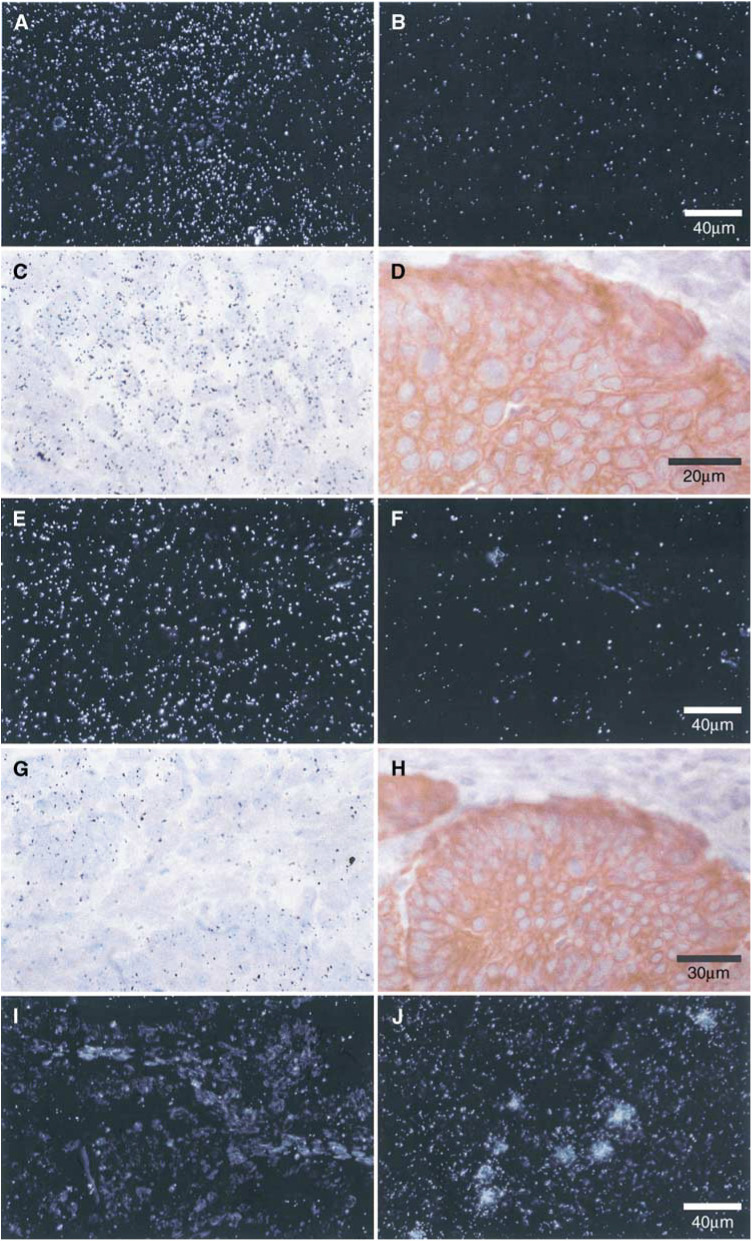
Expression of mRNA encoding VEGF-related factors in the epithelial cells of endometrioid endometrial carcinoma and ACH. *In situ* hybridisation with VEGF-A antisense probe in a moderately differentiated (FIGO grade 2) carcinoma shown under dark-field (**A**) and light-field (**C**) conditions shows hybridisation in epithelial carcinoma cells. There is no specific hybridisation when a sense probe (negative control) is used (**B**). The scale bar in (**B**) applies to (**A**), (**B**), (**E**), (**F**), (**I**) and (**J**). Immunostaining of a serial section with anti-human cytokeratin confirms the epithelial localisation of silver grains (**D**) (scale bar applies to ACH (**C**) and (**D**). *In situ* hybridisation with VEGF-B antisense probe in ACH shown under dark-field (**E**) and light-field (**G**) conditions shows diffuse hybridisation in epithelial and stromal cells. There is no specific hybridisation when a sense probe (negative control) is used (**F**). Immunostaining with anti-human cytokeratin (**H**) identifies the epithelial and stromal cells (**H**) (scale bar applies to (**G**) and (**H**). *In situ* hybridisation with VEGF-C antisense probe in a poorly differentiated (FIGO grade 3) carcinoma shown under dark-field conditions (**I**) shows no specific hybridisation when compared with a human B-cell lymphoma (**J**).

) and was increased near areas of necrosis. Hybridisation for VEGF-A was not seen in cases of ACH or in sections of benign postmenopausal endometrium.

In contrast, hybridisation of the VEGF-B riboprobe was seen in all the five cases of ACH (Table 2 and Figure 2). There was continued expression for VEGF-B in low-grade tumours that diminished as the grade of the tumour increased. Vascular endothelial growth factor-B mRNA was also present in epithelial and stromal compartments (Figure 2), although, unlike VEGF-A, there was no apparent increase in hybridisation near areas of necrosis.

Messenger RNA encoding VEGF-C was not detected in any of the tissues examined using *in situ* hybridisation (Table 2 and Figure 1). A specimen of human lymphoma was used as a positive control and demonstrated specific hybridisation (Figure 2). However, PCR amplification of VEGF-C cDNA was seen in all the samples of endometrial cancer (13 out of 13) and ACH (three out of three) examined (Figure 1), in addition to six out of 7 benign postmenopausal endometria. These findings confirm the presence of VEGF-C in benign, premalignant and malignant endometrium (Hirai *et al*, 2001), although at levels beneath the detection threshold for *in situ* hybridisation.

**Figure 3:**
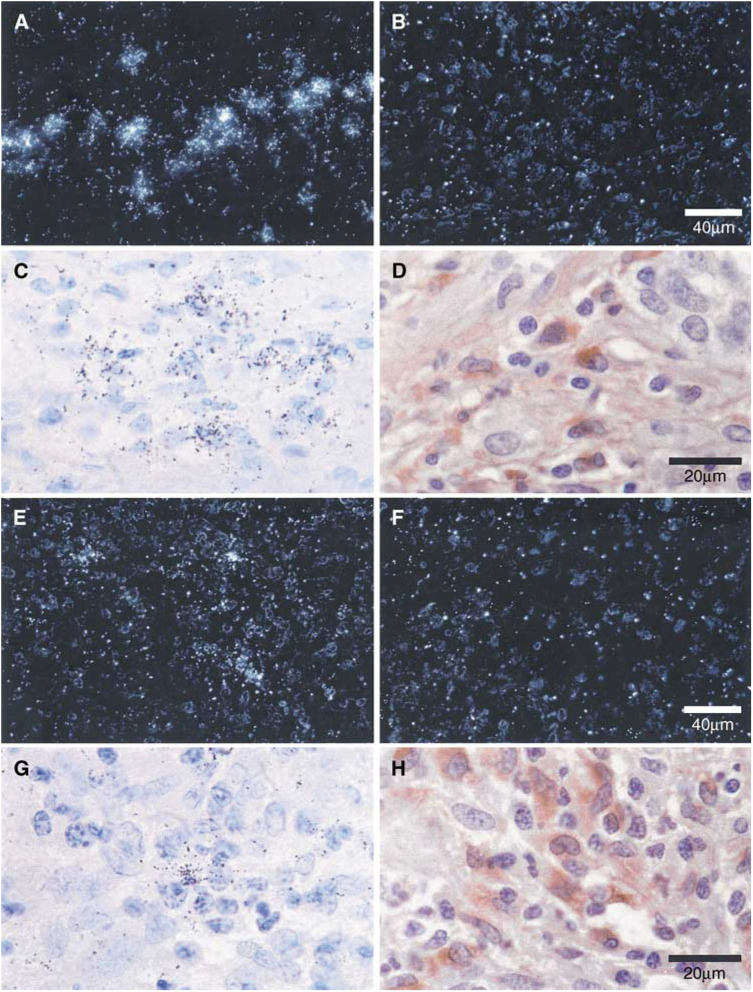
Expression of mRNA encoding VEGF-D and Ang-2 in macrophages infiltrating a poorly differentiated (FIGO grade 3) endometrioid endometrial carcinoma. *In situ* hybridisation with VEGF-D antisense probe shown under dark-field (**A**) conditions and light-field conditions (**C**). *In situ* hybridisation with VEGF-D sense (control) probe shows no specific hybridisation (**B**) (scale bar applies to (**A**), (**B**), (**E**) and (**F**)). Immunostaining with anti-human CD68 in a serial section (**D**), localises the silver grains to tumour associated macrophages (TAMs) (scale bar applies to (**C**), (**D**), (**G**) and (**H**)). *In situ* hybridisation with Ang-2 antisense probe shown under dark-field (**E**) and light-field (**G**) conditions. *In situ* hybridisation with Ang-2 sense (control) probe (**F**) shows no specific hybridisation. Anti-human cytokeratin staining (**H**) localises the hybridisation to TAMs.

Messenger RNA encoding VEGF-D was present in three of the five ACH specimens and in six of the 13 carcinomas (one moderately differentiated tumour and five poorly differentiated tumours). The distribution of silver grains did not correspond with epithelial tumour cells but did co-localise with tumour-associated macrophages (CD-68 positive cells) in serial sections (Figure 3

).

Hybridisation for Ang-1 mRNA was seen in only one benign and one ACH specimen. Similarly, mRNA encoding Ang-2 was seen in no benign tissues and in only one of the five hyperplastic samples. In contrast, while hybridisation was absent in well/moderately differentiated cancers, hybridisation for Ang-2 mRNA was clearly demonstrated in four out of nine of the poorly differentiated tumours examined. Hybridisation was most marked in macrophage-rich regions of these tumours and was seen to correspond with tumour-associated macrophages (CD-68) upon immunostaining of serial sections (Figure 3). In all of the experiments described, there was no specific hybridisation when the sense probes were used on adjacent sections.

### Transcript abundance of VEGF-B and Ang-2 in benign endometrium and endometrial carcinoma assessed by quantitative PCR

**Figure 4:**
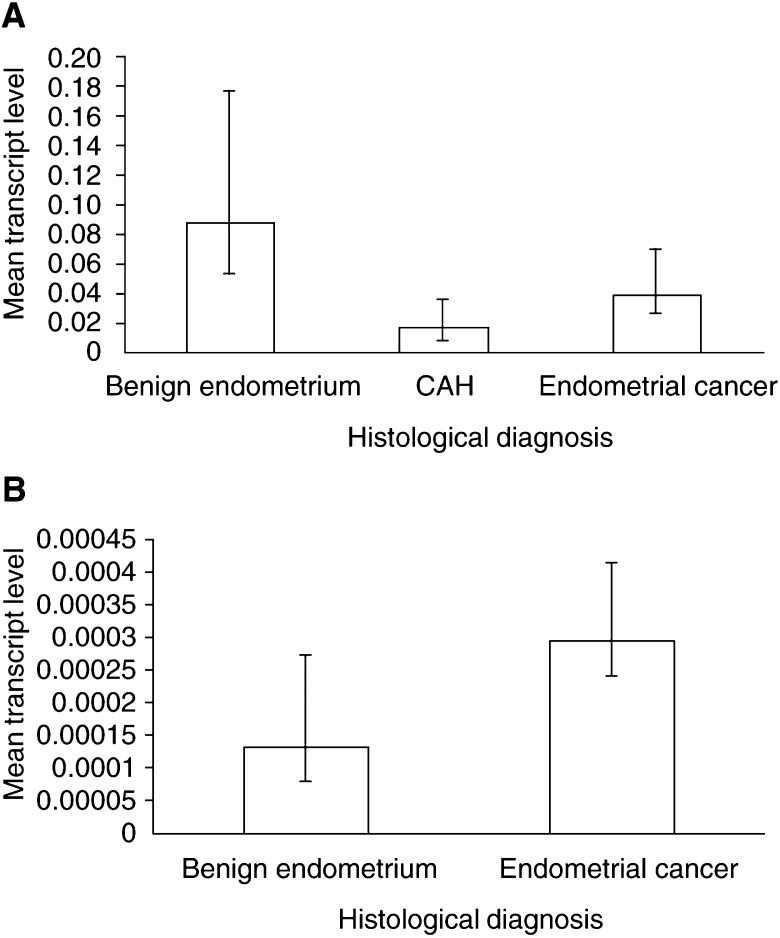
Quantitative PCR for gene transcripts in benign and malignant endometrium. Levels of gene transcript are shown normalised to an endogenous control sequence. Transcript levels are expressed as arbitrary units and represent the mean for each histological group. Experiments for each sample were performed in triplicate. (**A**) The level of mRNA encoding VEGF-B is generally higher in benign postmenopausal endometrium than in CAH (*P*=0.13) and endometrial cancer (*P*=0.04). (**B**) mRNA encoding Ang-2 is generally present at higher levels in endometrial cancer than in benign postmenopausal endometrium.

Real-time, quantitative PCR was carried out using cDNA generated from benign postmenopausal endometria and endometrioid-type endometrial adenocarcinoma. The abundance of VEGF-B transcript was greater in benign endometrium (*n*=7) than in atypical hyperplasia (*n*=3) (*P*=0.13) and endometrial cancer (*n*=17) (*P*=0.04) (Figure 4A

). Conversely, cDNA encoding Ang-2 was more abundant in the cancer samples (*n*=17) than in benign postmenopausal endometrium (*n*=4) (Figure 4B), although this did not reach statistical significance (*P*=0.49).

## DISCUSSION

These findings confirm the expression of VEGF-A in endometrial cancer (Doldi *et al*, 1996; Guidi *et al*, 1996; Fujimoto *et al*, 1998; Fine *et al*, 2000), although in contrast to other published reports (Fujimoto *et al*, 1998; Fine *et al*, 2000), we did not demonstrate VEGF-A mRNA in benign specimens. This may be explained by the inclusion of both pre- and postmenopausal endometrium in other studies (Fujimoto *et al*, 1998). Messenger RNA for VEGF-A is abundant in premenopausal endometrium (Charnock-Jones *et al*, 1993). Our findings support those of Guidi *et al* (1996), who demonstrated little or no mRNA expression in benign atrophic endometrium. We demonstrated VEGF-A mRNA in only one out of 5 specimens of ACH compared with 13 out of 13 endometrioid cancers. These differences may be due to necrosis within the malignant tissue. Hypoxia, in necrotic areas of cancer, leads to the upregulation of transcription (Tischer *et al*, 1991) and increased stability of VEGF-A mRNA (Levy *et al*, 1996).

Messenger RNA and protein for the related growth factor VEGF-B have previously been identified in other tumours including ovarian carcinoma (Sowter *et al*, 1997). We demonstrate, using quantitative PCR, that mRNA encoding VEGF-B is more abundant in benign postmenopausal endometrium than in endometrial adenocarcinoma. The results also suggest that there may be a relative loss of VEGF-B mRNA in ACH although this was not statistically significant and may reflect small sample numbers. We show, using *in situ* hybridisation, that where present, VEGF-B mRNA is expressed in both epithelium and stroma of ACH and some endometrial cancers. These findings contrast with the overexpression of VEGF-A seen in endometrial cancers (Doldi *et al*, 1996; Fujimoto *et al*, 1998). The distribution of VEGF-B in both epithelial and stromal compartments of atypical hyperplasia and some cancers is similar to that observed in ovarian cancer (Sowter *et al*, 1997), but in contrast to VEGF-A, hybridisation was not increased near areas of necrosis. Vascular endothelial growth factor-B is not upregulated by hypoxia (Silins *et al*, 1997) and this may explain the differences seen here.

The function of VEGF-B is not clear. Vascular endothelial growth factor-1 binds VEGFR-1 (flt-1) but not VEGFR-2 (KDR) (Olofsson *et al*, 1998), both of which are present in peritumoral endothelial cells of endometrial cancers (Guidi *et al*, 1996). Mouse ‘knockout’ experiments suggest that the VEGFR-1 plays a role in endothelial differentiation and/or interactions between the endothelium and extracellular matrix (Fong *et al*, 1995), whereas signalling through VEGFR-2 leads to endothelial cell proliferation and migration (Waltenberger *et al*, 1994). Vascular endothelial growth factor-B is therefore unlikely to promote endothelial cell proliferation or migration directly. However, VEGF-B signalling via VEGFR-1 may participate in the organisation of endothelial–matrix interactions as VEGF-B increases the expression of both uPA (urokinase-type plasminogen activator) and PAI-1 (plasminogen activator inhibitor-1) in endothelial cell cultures (Olofsson *et al*, 1996a). These play a role in matrix degradation and its inhibition, respectively.

Vascular endothelial growth factor-B could contribute to the maintenance of host–tumour responses. Vascular endothelial growth factor-A, produced by tumour cells, inhibits the functional maturation of immune dendritic cells from haemopoietic progenitor cells (Gabrilovich *et al*, 1996). This effect is mediated by binding to VEGFR-1 (Oyama *et al*, 1998). Therefore, loss of VEGF-B may contribute to the escape of tumour cells from the host immune response by making more VEGFR-1 binding sites available for binding by VEGF-A. Vascular endothelial growth factor-B may thus act to suppress changes that favour the development of endometrial cancer.

We confirmed the presence of VEGF-C mRNA in endometrial carcinoma shown by Hirai *et al* (2001), but did not demonstrate this by *in situ* hybridisation. Intense hybridisation for VEGF-C mRNA is seen in endometrial natural killer cells (Li *et al*, 2001), hence the VEGF-C transcript may be present in non-tumour cells that were under-represented in our endometrial tissues.

Vascular endothelial growth factor-D binds to VEGFR-2 and VEGFR-3 (Achen *et al*, 1998), both of which are present in endometrial cancer (Guidi *et al*, 1996). In colon cancer, VEGFR-3 is associated with inflammatory infiltrates of TAMs (White *et al*, 2002) although VEGF-D has not been demonstrated in TAMs themselves. We have shown hybridisation for VEGF-D mRNA in ACH and both well differentiated and poorly differentiated tumours (Table 2), although not all cases in each group were positive. Recently, positive immunostaining for VEGF-D has been demonstrated in both epithelium and stroma of endometrial cancers with some cases of ACH also displaying immunopositivity (Yokoyama *et al*, 2003). In the latter study, 42 and 87% of stage I/II and stage III/IV tumours, respectively, showed significant immunostaining. These findings may reflect differences in the degree of inflammatory infiltration between individual tumours as well as increased expression of VEGF-D by the tumour cells. There is a tendency to higher TAM density in microvessel-rich tumours (Salvesen and Akslen, 1999), and our findings indicate that TAMs may assist in promoting angiogenesis in endometrioid endometrial carcinoma by the secretion of VEGF-D.

Angiopoietin-1 and Ang-2 are previously unreported in endometrial cancer. We demonstrated higher levels of Ang-2 mRNA in endometrial cancers than benign endometrium, although this did not reach statistical significance. The latter may reflect the small numbers of samples in the benign group. Nonetheless, the distribution of mRNA for Ang-2, assessed by *in situ* hybridisation, was similar to that of VEGF-D in TAMs (Figure 3). Both Ang-2 and VEGF-D were only seen in association with CD68-positive cells, although CD68-positive cells, without hybridisation for either of these factors were also present.

Angiopoietin-2 is an antagonist of Ang-1, acting through the Tie-2 receptor and disrupting pericyte-endothelial cell interactions. In the presence of VEGF-A, Ang-2 facilitates vessel sprouting while in its absence, regression of vessel sprouts occurs (Maisonpierre *et al*, 1997). However, in active tumour angiogenesis, a large proportion of the blood vessels lack pericytes and can be selectively ablated by withdrawal of VEGF-A alone (Benjamin *et al*, 1999). In our series, survival did not differ significantly where hybridisation for both VEGF-A and Ang-2 was seen and this may reflect a high proportion of immature tumour vessels lacking pericyte coverage. Angiopoietin-2 may also alter the response of destabilised endothelial cells to cytokines, thus modulating angiogenesis (Laurén *et al*, 1998).

We have shown that gene transcripts for the related growth factors, VEGF-A and VEGF-B, are differentially expressed in benign postmenopausal endometrium and endometrioid endometrial cancer. The reduced expression of VEGF-B in endometrial cancers compared with benign endometrium suggests that VEGF-B may play a role in maintaining normal cellular interactions in endometrium and that loss of expression could contribute to endometrial tumorigenesis.

## References

[bib1] Abu-Jawdeh GM, Faix JD, Niloff J, Tognazzi K, Manseau E, Dvorak HF, Brown LF (1996) Strong expression of vascular permeability factor (vascular endothelial growth factor) and its receptors in ovarian borderline and malignant neoplasms. Lab Invest 74: 1105–1115 8667614

[bib2] Abulafia O, Triest WE, Sherer DM, Hansen CC, Ghezzi F (1995) Angiogenesis in endometrial hyperplasia and stage I endometrial carcinoma. Obstet Gynecol 86: 479–485 7545803 10.1016/0029-7844(95)00203-4

[bib3] Achen MG, Jeltsch M, Kukk E, Makinen T, Vitali A, Wilks AF, Alitalo K, Stacker SA (1998) Vascular endothelial growth factor D (VEGF-D) is a ligand for the tyrosine kinases VEGF receptor 2 (Flk1) and VEGF receptor 3 (Flt4). Proc Natl Acad Sci USA 95: 548–553 9435229 10.1073/pnas.95.2.548 PMC18457

[bib4] Benjamin LE, Golijanin D, Itin A, Pode D, Keshet E (1999) Selective ablation of immature blood vessels in established human tumors follows vascular endothelial growth factor withdrawal. J Clin Invest 103: 159–165 9916127 10.1172/JCI5028 PMC407882

[bib5] Boocock CA, Charnock-Jones DS, Sharkey AM, McLaren J, Barker PJ, Wright KA, Twentyman PR, Smith SK (1995) Expression of vascular endothelial growth factor and its receptors flt and KDR in ovarian carcinoma. J Natl Cancer Inst 87: 506–516 7707437 10.1093/jnci/87.7.506

[bib6] Charnock-Jones DS, Sharkey AM, Rajput-Williams J, Burch D, Schofield JP, Fountain SA, Boocock CA, Smith SK (1993) Identification and localization of alternately spliced mRNAs for vascular endothelial growth factor in human uterus and estrogen regulation in endometrial carcinoma cell lines. Biol Reprod 48: 1120–1128 8481475 10.1095/biolreprod48.5.1120

[bib7] Chomczynski P, Sacchi N (1987) Single-step method of RNA isolation by acid guanidinium thiocyanate–phenol–chloroform extraction. Anal Biochem 162: 156–159 2440339 10.1006/abio.1987.9999

[bib8] Clark DE, Smith SK, Sharkey AM, Sowter HM, Charnock-Jones DS (1996) Hepatocyte growth factor /scatter factor and its receptor c-met: localisation and expression in the human placenta throughout pregnancy. J Endocrinol 151: 459–467 8994391 10.1677/joe.0.1510459

[bib9] Davis S, Aldrich TH, Jones PF, Acheson A, Compton DL, Jain V, Ryan TE, Bruno J, Radziejewski C, Maisonpierre PC, Yancopoulos GD (1996) Isolation of angiopoietin-1, a ligand for the TIE2 receptor, by secretion-trap expression cloning. Cell 87: 1161–1169 8980223 10.1016/s0092-8674(00)81812-7

[bib10] Doldi N, Bassan M, Gulisano M, Broccoli V, Boncinelli E, Ferrari A (1996) Vascular endothelial growth factor messenger ribonucleic acid expression in human ovarian and endometrial cancer. Gynecol Endocrinol 10: 375–382 9032563 10.3109/09513599609023600

[bib11] Ferrara N, Carver-Moore K, Chen H, Dowd M, Lu L, O'Shea KS, Powell-Braxton L, Hillan KJ, Moore MW (1996) Heterozygous embryonic lethality induced by targeted inactivation of the VEGF gene. Nature 380: 439–442 8602242 10.1038/380439a0

[bib12] Fine BA, Valente PT, Feinstein GI, Dey T (2000) VEGF, flt-1, and KDR/flk-1 as prognostic indicators in endometrial carcinoma. Gynecol Oncol 76: 33–39 10620438 10.1006/gyno.1999.5658

[bib13] Folkman J (1990) What is the evidence that tumors are angiogenesis dependent? J Natl Cancer Inst 82: 4–6 1688381 10.1093/jnci/82.1.4

[bib14] Fong GH, Rossant J, Gertsenstein M, Breitman ML (1995) Role of the Flt-1 receptor tyrosine kinase in regulating the assembly of vascular endothelium. Nature 376: 66–70 7596436 10.1038/376066a0

[bib15] Fujimoto J, Ichigo S, Hirose R, Sakaguchi H, Tamaya T (1998) Expressions of vascular endothelial growth factor (VEGF) and its mRNA in uterine endometrial cancers. Cancer Lett 134: 15–22 10381125 10.1016/s0304-3835(98)00232-8

[bib16] Gabrilovich DI, Chen HL, Girgis KR, Cunningham HT, Meny GM, Nadaf S, Kavanaugh D, Carbone DP (1996) Production of vascular endothelial growth factor by human tumors inhibits the functional maturation of dendritic cells. Nat Med 2: 1096–1103 8837607 10.1038/nm1096-1096

[bib17] Goede V, Schmidt T, Kimmina S, Kozian D, Augustin HG (1998) Analysis of blood vessel maturation processes during cyclic ovarian angiogenesis. Lab Invest 78: 1385–1394 9840613

[bib18] Guidi AJ, Abu-Jawdeh G, Tognazzi K, Dvorak HF, Brown LF (1996) Expression of vascular permeability factor (vascular endothelial growth factor) and its receptors in endometrial carcinoma. Cancer 78: 454–460 8697391 10.1002/(SICI)1097-0142(19960801)78:3<454::AID-CNCR12>3.0.CO;2-Y

[bib19] Hanahan D (1997) Signaling vascular morphogenesis and maintenance. Science 277: 48–50 9229772 10.1126/science.277.5322.48

[bib20] Hirai M, Nakagawara A, Oosaki T, Hayashi Y, Hirono M, Yoshihara T (2001) Expression of vascular endothelial growth factors (VEGF-A/VEGF-1 and VEGF-C/VEGF-2) in postmenopausal uterine endometrial carcinoma. Gynecol Oncol 80: 181–188 11161857 10.1006/gyno.2000.6056

[bib21] Holash J, Maisonpierre PC, Compton D, Boland P, Alexander CR, Zagzag D, Yancopoulos GD, Wiegand SJ (1999) Vessel cooption, regression, and growth in tumors mediated by angiopoietins and VEGF. Science 284: 1994–1998 10373119 10.1126/science.284.5422.1994

[bib22] Houck KA, Ferrara N, Winer J, Cachianes G, Li B, Leung DW (1991) The vascular endothelial growth factor family: identification of a fourth molecular species and characterization of alternative splicing of RNA. Mol Endocrinol 5: 1806–1814 1791831 10.1210/mend-5-12-1806

[bib23] Joukov V, Pajusola K, Kaipainen A, Chilov D, Lahtinen I, Kukk E, Saksela O, Kalkkinen N, Alitalo K (1996) A novel vascular endothelial growth factor, VEGF-C, is a ligand for the Flt4 (VEGFR-3) and KDR (VEGFR-2) receptor tyrosine kinases. EMBO J 15: 1751 8612600 PMC450088

[bib24] Kaku T, Kamura T, Kinukawa N, Kobayashi H, Sakai K, Tsuruchi N, Saito T, Kawauchi S, Tsuneyoshi M, Nakano H (1997) Angiogenesis in endometrial carcinoma. Cancer 80: 741–747 9264358 10.1002/(sici)1097-0142(19970815)80:4<741::aid-cncr13>3.0.co;2-t

[bib26] Lauren J, Gunji Y, Alitalo K (1998) Is angiopoietin-2 necessary for the initiation of tumor angiogenesis? Am J Pathol 153: 1333–1339 9811321 10.1016/S0002-9440(10)65717-3 PMC1853422

[bib27] Levy AP, Levy NS, Goldberg MA (1996) Post-transcriptional regulation of vascular endothelial growth factor by hypoxia. J Biol Chem 271: 2746–2753 8576250 10.1074/jbc.271.5.2746

[bib28] Li XF, Charnock-Jones DS, Zhang E, Hiby S, Malik S, Day K, Licence D, Bowen JM, Gardner L, King A, Loke YW, Smith SK (2001) Angiogenic growth factor messenger ribonucleic acids in uterine natural killer cells. J Clin Endocrinol Metab 86: 1823–1834 11297624 10.1210/jcem.86.4.7418

[bib29] Maisonpierre PC, Suri C, Jones PF, Bartunkova S, Wiegand SJ, Radziejewski C, Compton D, McClain J, Aldrich TH, Papadopoulos N, Daly TJ, Davis S, Sato TN, Yancopoulos GD (1997) Angiopoietin-2, a natural antagonist for Tie2 that disrupts *in vivo* angiogenesis. Science 277: 55–60 9204896 10.1126/science.277.5322.55

[bib30] Millauer B, Shawver LK, Plate KH, Risau W, Ullrich A (1994) Glioblastoma growth inhibited *in vivo* by a dominant-negative Flk-1 mutant. Nature 367: 576–579 8107827 10.1038/367576a0

[bib31] Ohta Y, Shridhar V, Bright RK, Kalemkerian GP, Du W, Carbone M, Watanabe Y, Pass HI (1999) VEGF and VEGF type C play an important role in angiogenesis and lymphangiogenesis in human malignant mesothelioma tumours. Br J Cancer 81: 54–61 10487612 10.1038/sj.bjc.6690650 PMC2374345

[bib35] Olofsson B, Korpelainen E, Pepper MS, Mandriota SJ, Aase K, Kumar V, Gunji Y, Jeltsch MM, Shibuya M, Alitalo K, Eriksson U (1998) Vascular endothelial growth factor B (VEGF-B) binds to VEGF receptor-1 and regulates plasminogen activator activity in endothelial cells. Proc Natl Acad Sci USA 95: 11709–11714 9751730 10.1073/pnas.95.20.11709 PMC21705

[bib32] Olofsson B, Pajusola K, Kaipainen A, von Euler G, Joukov V, Saksela O, Orpana A, Pettersson RF, Alitalo K, Eriksson U (1996a) Vascular endothelial growth factor B, a novel growth factor for endothelial cells. Proc Natl Acad Sci USA 93: 2576–2581 8637916 10.1073/pnas.93.6.2576 PMC39839

[bib33] Olofsson B, Pajusola K, von Euler G, Chilov D, Alitalo K, Eriksson U (1996b) Genomic organization of the mouse and human genes for vascular endothelial growth factor B (VEGF-B) and characterization of a second splice isoform. J Biol Chem 271: 19310–19317 8702615 10.1074/jbc.271.32.19310

[bib36] Olson TA, Mohanraj D, Carson LF, Ramakrishnan S (1994) Vascular permeability factor gene expression in normal and neoplastic human ovaries. Cancer Res 54: 276–280 8261452

[bib37] Olson TA, Mohanraj D, Carson LF, Ramakrishnan S (1996) *In vivo* neutralization of vascular endothelial growth factor (VEGF)/vascular permeability factor (VPF) inhibits ovarian carcinoma-associated ascites formation and tumor growth. Int J Oncol 8: 505–511 21544389 10.3892/ijo.8.3.505

[bib38] Oyama T, Ran S, Ishida T, Nadaf S, Kerr L, Carbone DP, Gabrilovich DI (1998) Vascular endothelial growth factor affects dendritic cell maturation through the inhibition of nuclear factor-kappa B activation in hemopoietic progenitor cells. J Immunol 160: 1224–1232 9570538

[bib39] Salvesen HB, Akslen LA (1999) Significance of tumour-associated macrophages, vascular endothelial growth factor and thrombospondin-1 expression for tumour angiogenesis and prognosis in endometrial carcinomas. Int J Cancer 84: 538–543 10502735 10.1002/(sici)1097-0215(19991022)84:5<538::aid-ijc17>3.0.co;2-b

[bib40] Shalaby F, Rossant J, Yamaguchi TP, Gertsenstein M, Wu XF, Breitman ML, Schuh AC (1995) Failure of blood-island formation and vasculogenesis in Flk-1-deficient mice. Nature 376: 62–66 7596435 10.1038/376062a0

[bib41] Silins G, Grimmond S, Egerton M, Hayward N (1997) Analysis of the promoter region of the human VEGF-related factor gene. Biochem Biophys Res Commun 230: 413–418 9016794 10.1006/bbrc.1996.5979

[bib42] Sowter HM, Corps AN, Evans AL, Clark DE, Charnock-Jones DS, Smith SK (1997) Expression and localization of the vascular endothelial growth factor family in ovarian epithelial tumors. Lab Invest 77: 607–614 9426398

[bib43] Thurston G, Suri C, Smith K, McClain J, Sato TN, Yancopoulos GD, McDonald DM (1999) Leakage-resistant blood vessels in mice transgenically overexpressing angiopoietin-1. Science 286: 2511–2514 10617467 10.1126/science.286.5449.2511

[bib44] Tischer E, Mitchell R, Hartman T, Silva M, Gospodarowicz D, Fiddes JC, Abraham JA (1991) The human gene for vascular endothelial growth factor. Multiple protein forms are encoded through alternative exon splicing. J Biol Chem 266: 11947–11954 1711045

[bib45] Waltenberger J, Claesson-Welsh L, Siegbahn A, Shibuya M, Heldin CH (1994) Different signal transduction properties of KDR and Flt1, two receptors for vascular endothelial growth factor. J Biol Chem 269: 26988–26995 7929439

[bib46] White JD, Hewett PW, Kosuge D, McCulloch T, Enholm BC, Carmichael J, Murray JC (2002) Vascular endothelial growth factor-D expression is an independent prognostic marker for survival in colorectal carcinoma. Cancer Res 62: 1669–1675 11912138

[bib47] Yokoyama Y, Charnock-Jones DS, Licence D, Yanaihara A, Hastings JM, Holland CM, Emoto M, Sakamoto T, Maruyama, H, Sato S, Mizunuma H, Smith SK (2003) Expression of vascular endothelial growth factor (VEGF)-D and its receptor, VEGF Receptor 3, as a prognostic indicator in endometrial carcinoma. Clin Cancer Res 9: 1361–1369 12684405

